# Protective Effect of Curcumin against Sodium Salicylate-Induced Oxidative Kidney Damage, Nuclear Factor-Kappa Dysregulation, and Apoptotic Consequences in Rats

**DOI:** 10.3390/antiox10060826

**Published:** 2021-05-21

**Authors:** Yasmina M. Abd-Elhakim, Attia A. A. Moselhy, Adil Aldhahrani, Rasha R. Beheiry, Wafaa A. M. Mohamed, Mohamed Mohamed Soliman, Bayan A. Saffaf, Maha M. El Deib

**Affiliations:** 1 Department of Forensic Medicine and Toxicology, Faculty of Veterinary Medicine, Zagazig University, Zagazig 44519, Egypt; 2 Department of Anatomy and Embryology, Faculty of Veterinary Medicine, Zagazig University, Zagazig 44519, Egypt; atiaanatomy@gmail.com; 3 Clinical Laboratory Sciences Department, Turabah University College, Taif University, Turabah 21995, Saudi Arabia; a.ahdhahrani@tu.edu.sa (A.A.); mmsoliman@tu.edu.sa (M.M.S.); 4 Department of Histology and Cytology, Faculty of Veterinary Medicine, Zagazig University, Zagazig 44519, Egypt; rasharagab2006@yahoo.com; 5 Department of Clinical Pathology, Faculty of Veterinary Medicine, Zagazig University, Zagazig 44519, Egypt; waffa.clinical@yahoo.com; 6 Pharmacology Department, Faculty of Pharmacy, Future University, City of the Future 41639, Egypt; Bayan.saffaf@fue.edu.eg; 7 Department of Biochemistry, Faculty of Veterinary Medicine, Zagazig University, Zagazig 44519, Egypt; dr.mmeldeib@yahoo.com

**Keywords:** curcumin, sodium salicylate, kidney, anemia, NF-kappa B, Caspase-3

## Abstract

This study examined the effect of sodium salicylates (SS), alone and in combination with curcumin (CUR), on kidney function and architecture in rats. Five rat groups were given 1 mL physiological saline/rat orally, 1 mL olive oil/rat orally, 50 mg CUR/kg bwt orally, 300 mg SS/kg bwt intraperitoneally, or CUR+SS for 15 days. The hematological indices, serum protein profile, serum electrolytes balance, oxidative stress, and lipid peroxidation of kidney tissues were assessed. The histopathological examination and immune expression of Caspase-3 and nuclear factor kappa (NF-κB) were conducted. The findings showed that SS injection induced nephrotoxic activity, including increased serum urea, creatinine, and uric acid levels. It also caused apparent pathological alterations with increased Caspase-3 and NF-κB immuno-expression. In addition, thrombocytopenia, leukocytosis, neutrophilia, hyponatremia, hypochloremia, hypocalcemia, and hypomagnesemia but not hyperkalemia and hyperphosphatemia were evident in SS-injected rats. Moreover, SS exposure increased serum α1 globulin, renal tissue malondialdehyde, and Caspase-3 levels but superoxide dismutase, glutathione peroxidase, and Bcl-2 levels declined. Meanwhile, CUR significantly counteracted the SS harmful impacts on kidneys but SS+CUR co-administration induced an anemic condition. Overall, CUR has an evident protective role against SS-induced renal damage, but the disturbed hematological alterations should be carefully taken into consideration in their combined use.

## 1. Introduction

Sodium salicylate (SS) is commonly prescribed as an anti-inflammatory and pain relief drug [1]. Despite the important therapeutic roles of SS, several complications have been reported to be associated with SS overdose, including tinnitus [2], hearing loss [3], and neurotoxicity [4]. Furthermore, a high single salicylates dose in rats led to increased urinary levels of enzymes coming from proximal tubular cells. These results were often linked to biochemical indices of proximal tubular dysfunction such as potassium wastage and glucosuria [5,6]. In most cases, proximal tubular dysfunction caused by salicylate occurred within several hours of administration and rarely lasted longer than several days. Morphological deviations associated with compromised tubular function ranged from mild to deep proximal tubular necrosis with the extension of nuclear pyknosis and karyolysis and with proximal membrane degeneration. Tubular epithelial regeneration was not observed until 24 h following treatment [7]. Additionally, deliberate ingestion or unintentional overdose of SS can result in severe metabolic derangements, making treatment challenging. Moreover, co-ingestion of other medications can further complicate management [8]. Therefore, complementary medicinal products can be a worthwhile approach to address the above challenges, adding to SS effectiveness while masking toxicity [9].

In recent years, several natural products showed strong nephroprotective activity [10,11,12,13]. Curcumin (CUR), the *Curcuma longa Linn* yellow pigment, has attracted much attention for medicinal purposes in a wide range of illnesses such as autoimmune diseases [14], cancer [15], diabetes mellitus [16], and fatty liver disease [17,18]. CUR has a potent antioxidant [19,20], anti-inflammatory [14], antiproliferative [21,22], immunostimulant [23], and neuroprotective activities [4,24]. In addition, several recent reports have confirmed the protective role of CUR against drug-induced nephrotoxicity [25,26,27]. Recently, Mohapatra, et al. [9] developed a co-amorphous mixture of acetylsalicylic acid and CUR for improving dissolution, enhancing anti-inflammatory activity, and diminishing gastro toxicity. They confirmed that this low-dose combination could be a successful approach for anti-inflammation combination therapy. On the other hand, some reports spotlighted the dark aspects of CUR, particularly in combination with certain medications or drugs [28]. For instance, Khalaji, et al. [29] reported that the combined use of CUR with a derivative of amphetamine named ecstasy induced serious consequences on hematological parameters and serum immunoglobin levels.

CUR showed nephroprotective effects when orally dosed at 50 mg/kg bwt against xenobiotic-inducing kidney damage [30,31,32]. Hence, in the current study, we explored the outcomes of CUR (50 mg/kg bwt) co-treatment with large doses of SS (300 mg/kg bwt) on blood cells and kidney function. For this purpose, rats were given CUR and/or SS for 15 days and then exposed to hematological, biochemical, histopathological, and immunohistochemical evaluations.

## 2. Materials and Methods

### 2.1. Tested Compounds and Chemicals

SS from El Nasr pharmaceutical chemicals “Adwic”, Cairo, Egypt, was used. A stock solution of CUR was prepared using olive oil (Colavita via laurentina”, Rome, Italy). All other chemicals were purchased from Sigma-Aldrich Co., St. Louis, MO, USA.

### 2.2. Animals and Experimental Design

Fifty healthy Sprague Dawley rats (male, 140–160 g, 10 weeks of age) were attained from the Laboratory Animal Housing Unit, Faculty of Veterinary Medicine, Zagazig University, Egypt. In a 12 h light/12 h dark, rats are kept in a stainless steel cage with free, accessible food and water in a well-ventilated room. Two weeks before any experimental studies were carried out, the experimental animals were acclimated to laboratory conditions. Randomly five groups (10 rats/treatment) were divided between the experimental group and control: control, orally administered 1 mL of saline /rat; olive oil group (OO), orally given 1 mL of olive oil/rat; CUR group, orally given 50 mg of CUR /kg bwt [33]; SS group, intraperitoneally injected a dose of 300 mg/kg bwt of SS in bacteriostatic saline 60 mg/mL once daily [34]; and CUR+SS group, CUR and SS were administered at the doses and routes mentioned above. The experiment continued for 15 days, as several documented case studies showed various side effects in patients who consumed SS up to 15 successive days [35]. Additionally, in this context, Yi, et al. [36] assessed the SS side effects when orally administered at a dose of 200 mg/kg bwt for 14 consecutive days. The pain, injury, abnormal behavior, distress, mucous membranes, breathing patterns, morbidity, and mortality during the experiment were closely monitored.

### 2.3. Sampling

At the end of the experiment, the rats were anesthetized and then euthanized by decapitation. Blood samples were collected from the retro-orbital plexus. For hematological analysis, the samples were collected in tubes with EDTA 10% as an anticoagulant. The other samples were taken in plain tubes and were left to clot for 30 min at room temperature. Then, the samples were centrifuged at 3000 rpm for 20 min. The resultant serum was stored at −20 °C until analysis. The kidneys were removed, saline-washed, weighed, and then divided into two groups of specimens. The first group was homogenized and centrifuged at 664× *g* for 15 min at 4 °C, and the resultant supernatants were used in the biochemical indicator estimation. For histopathological and immunohistopathological examinations, the second group was stored in 10% neutral buffered formalin.

### 2.4. Hematological Analysis

Complete blood counts, including leukogram and erythrogram profiles, were measured using an automated blood cell analyzer (Hemascreen18, Hospitex diagnostics, Sesto Fiorentino, Italy).

### 2.5. Serum Biochemical Analysis

#### 2.5.1. Measurement of Some Kidney Function Tests

The protocols by Coulombe and Favreau [37], and Larsen [38] were used to assess the serum levels of urea and creatinine, respectively. The concentration of uric acid was estimated using the technique by Barham and Trinder [39]. The potassium tetraphenylborate gravimetric method without deproteinization [40] and the single reagent method [41] were used to detect potassium (K) and sodium (Na) amounts, respectively. Magnesium (Mg), calcium (Ca), chloride (Cl), and phosphorous (Ph) were determined colorimetrically using bio-diagnostic kits (Giza, Egypt) following the methods of Tietz [42], Gindler and King [43], Schales and Schales [44], and El-Merzabani and El-Aaser [45], respectively.

#### 2.5.2. Protein Profile

The total level of serum protein was estimated according to the method of Gornal, et al. [46]. Based on the technique of Doumas, et al. [47], serum albumin concentration was evaluated. By subtracting the albumin amount from the total protein amount, total amount of globulin was calculated [48]. Serum protein fractionation was performed using sodium sulfate-polyacrylamide gel electrophoresis (SDS-PAGE) technique for the determination of serum alpha (α), beta (β), and gamma (ɣ) globulins according to the method illustrated by Ornstein [49].

### 2.6. Assessment of Oxidative Stress and Apoptotic Parameters in Kidney Tissue

The methods applied by Nishikimi, et al. [50] and by Paglia and Valentine [51] were used for the evaluation of superoxide dismutase (SOD) and glutathione peroxidase (GPx), respectively, using commercial colorimetric bioassay kits (Biodiagnostic Co. Dokki, Giza, Egypt). A lipid peroxidation biomarker, malondialdehyde (MDA) content, was measured by the Ohkawa, et al. [52] method.

Rat ELISA kits were used to detect apoptotic indicators in the kidney tissue, comprising Caspase-3 and Bcl-2. The kits were obtained from MyBioSource (San Diego, CA, USA) (catalog No. MBS700575 and MBS2515143) for Caspase-3 and Bcl-2, respectively. The quantification was carried out in triples according to the instructions of the manufacturer.

### 2.7. Histopathological Investigations

The collected kidney tissue specimens were kept for 48 h in 10% neutral buffered formalin. Then, formalin-fixed specimens were dehydrated in graded ethanol (70–100%), cleaned with xylene for one hour two times, and embedded in paraffin. The paraffinized blocks were then divided into 5 micron sections and stained with hematoxylin and eosin [53].

### 2.8. Immunohistochemical Investigation of Caspase-3 and NF-κB Activity in the Kidney Tissues

Another set of kidney paraffin sections was used for Caspase-3 detection by a rabbit polyclonal antibody (cat no: RB-1197-R7 Thermo Fisher Scientific, Waltham, MA, USA). For NF-κB investigation, some kidney paraffin sections were used, stained for NF-κB by rabbit polyclonal NF-κB p65 (phospho S276) primary antibody (ab194726), goat anti-rabbit IgG H&L (HRP) secondary antibody (ab205718) (Abcam, Cambridge, UK), and 3,3′-diaminobenzidine chromogen in line with the ABC method [54]

### 2.9. Data Analysis

The computer program SPSS/PC +2001 was used for statistical analysis of the study data. The statistical method used was one-way ANOVA and Dunnett’s multiple comparison test. The data are displayed as mean ± SD. A minimum significance level of *p* < 0.05 was set.

## 3. Results

### 3.1. Effects on Relative Kidney Weights and Hematological Indicators

As shown in Table 1, no significant differences were recorded in the right or left relative kidney weights among the different experimental groups. Neither the individual SS injection nor the oral CUR doses alter the erythrogram elements compared to the control groups. However, the combined SS+CUR administration significantly (*p* < 0.001) reduced the RBC count, Hb content, PCV%, and MCH compared to the other experimental groups. However, no significant changes in MCV and MCHC were recorded among different experimental groups. Alternatively, a significant (*p* < 0.001) decline in the platelet count was recorded in the SS-injected rats compared to the control group. Conversely, the CUR and CUR+SS-treated groups showed a significant increase in the platelet count compared to the SS-injected and control groups.

Regarding the leukogram, the SS-injected group displayed a significant increase in the WBCs (*p* < 0.001) and neutrophil (*p* = 0.061) counts relative to the control groups. In contrast, the CUR oral dose significantly suppressed the SS-induced increments in the number of WBCs and neutrophils compared to the SS-injected groups to a level nonsignificantly different from the control group. Nonetheless, no significant changes in the counts of lymphocytes, monocytes, eosinophils, and basophils were found among the diverse experimental groups.

### 3.2. Effects on Protein Fractions

The influence of a CUR oral dose for 15 days and/or SS intraperitoneal injection in rats on protein profile is revealed in Table 2. The SS-injected rats showed a significant (*p* < 0.001) reduction in the total protein, albumin, total globulin, and γ globulin by 34.91%, 43.44%, 24.63%, and 55.47% relative to the control group. However, a significant (*p* < 0.001) increase in α2glob was found in the SS-injected rats by 34.19% compared to the control group. On the other hand, SS-induced depletion in the level of total protein, albumin, total globulin, and γ globulin was restored less than the control group by 20.68%, 24.75%, 15.78%, and 35.94%, respectively, when combined with CUR oral administration.

Moreover, the SS-induced increment in α2glob amount was minimized with a concurrent CUR oral dose to 20.59% relative to the control group. Nevertheless, no significant alteration in the concentrations of α1 and β globulins was detected among different experimental groups.

### 3.3. Effects on Kidney Function Indicators

As demonstrated in Table 2, a significant (*p* < 0.001) increase in the level of kidney damage including urea, creatinine, and uric acid by 82.92%, 48.75%, and 105.16%, respectively, was recorded in the SS-injected rats relative to the control group. However, the CUR oral dose in SS-injected rats significantly depressed the urea, creatinine, and uric acid increments to 47.99%, 25.30%, and 53.69%, respectively.

### 3.4. Effects on Serum Electrolytes Levels

As revealed in Table 2, SS-injected rats showed a noticeable disturbance in the electrolyte balance, as demonstrated by a significant (*p* < 0.001) reduction in the levels of Na, Cl, Ca, and Mg by 35.11%, 28.11%, 53.57%, and 46.10%, respectively, relative to the control group. In contrast, a significant (*p* < 0.001) increment in the K and Ph was recorded in the SS-injected group by 149.36% and 95.98%, respectively, relative to the control group. Contrariwise, the CUR+SS treated group showed a significant correction of electrolyte levels relative to the SS-injected group.

### 3.5. Effects on Oxidative Kidney Damage

**Figure 1:**
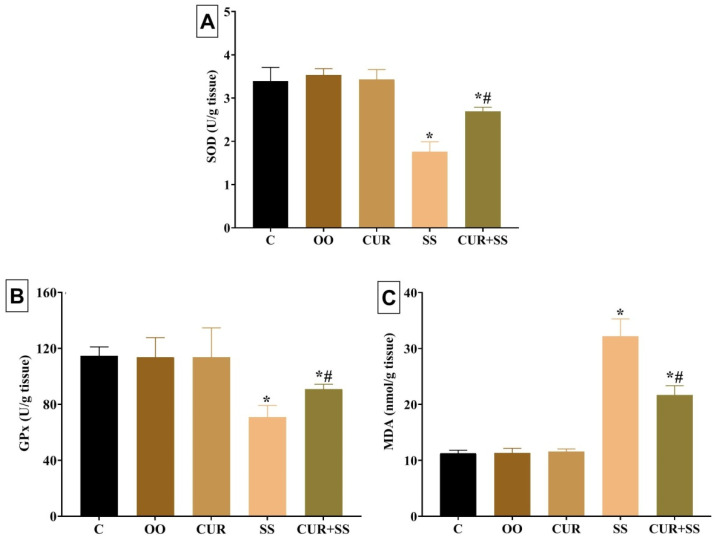
Effect of curcumin (CUR) oral dose on (**A**) superoxide dismutase (SOD), (**B**) glutathione peroxidase (GPx), and (**C**) malondialdehyde (MDA) in the kidney tissues of sodium salicylate (SS)-injected rats for 15 days. C: control group. OO: olive oil. Data are expressed as mean ± SD, *n* = 10 for each group. * Significantly different compared to the control groups at *p* < 0.05. ^#^ Significantly different from the SS-treated group at *p* < 0.05.

A significant (*p* < 0.001) exhaustion of SOD and GPx enzymes by 48.01% and 38.17%, respectively, but a significant elevation of MDA (twofold) were recorded in SS-injected rats relative to the control group (Figure 1). On the contrary, the SOD and GPx level in the CUR+SS-treated group was re-established at 20.65% and 20.68%, respectively, less than the control group. Furthermore, CUR significantly counteracted the SS-induced increase in the MDA content to one-fold in the CUR+SS-treated group more than the control group.

### 3.6. Effects on Apoptotic Indicators in Kidney Tissues

**Figure 2:**
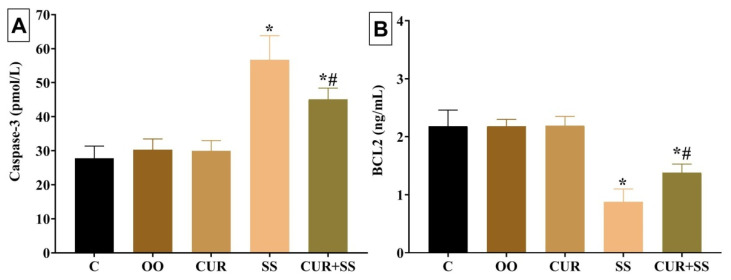
Effect of curcumin (CUR) oral dose on Caspase-3 (**A**) and BCL-2 (**B**) in the kidney tissues of sodium salicylate (SS)-injected rats for 15 days. C: control group. OO: olive oil. Data are expressed as mean ± SD, *n* = 10 for each group. * Significantly different compared to the control groups at *p* < 0.05. ^#^ Significantly different from the SS-treated group at *p* < 0.05.

As revealed in Figure 2, the apoptotic protein level of Caspase-3 was significantly (*p* < 0.001) augmented by 87.51% in SS-injected rats relative to the control group. Nonetheless, compared to the control group, SS injection for 15 days resulted in a significant (*p* < 0.001) decrease (59.86%) in the antiapoptotic protein Bcl-2. In the CUR+SS group, the SS-induced expression of the apoptotic protein Caspase-3 was reduced to 49.07% compared to the control group. Moreover, the SS-induced decline in the Bcl-2 level was restored by a CUR oral dose to 36.81% in the CUR+SS-treated group compared to the control group.

### 3.7. Histopathological Findings

**Figure 3:**
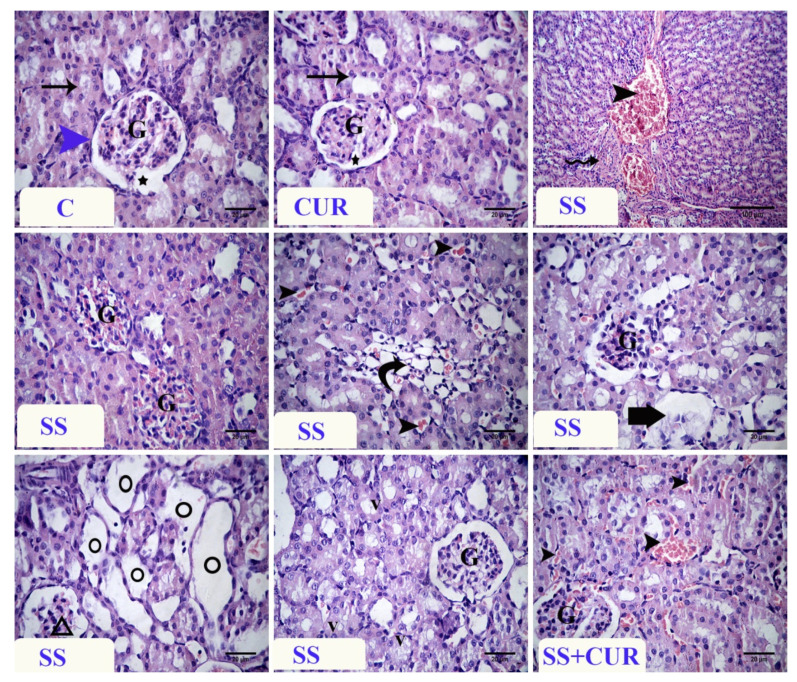
Photomicrograph of H&E-stained renal tissue sections. Control (**C**) and curcumin (CUR) group showing the normal glomeruli (G), renal tubules (arrows), outer layer of Bowman’s capsule (blue arrowhead), and capsular space (star). Sodium salicylate (SS)-treated group showing vascular congestion (arrowhead) and hyalinization of the blood vessel (zigzag arrow), absence of capsular space and glomerular congestion (G), coagulative necrosis (curved arrow), congestion of peritubular capillaries (arrowheads), absence of glomerular tuft (thick arrow), tubular dilation (circles) shrinkage of the glomerulus (triangle), and vacuolation of the lining epithelium of tubules (v). SS + CUR-treated group showing vascular congestion (arrowheads) and normal glomeruli (G). The magnification was ×400 in all photos, except the first one of SS was ×100.

Histological observations of the kidney of control and CUR-treated rats showed that the normal corpuscles formed from the outer and inner Bowman’s capsule, glomerulus and capsular space, and normal renal tubules. The SS-treated rats’ kidneys revealed degenerative changes in the renal tubules, vascular congestion, and severe glomerular congestion. The absence of the capsular space with severe congestion of the peritubular capillaries was noticed. Moreover, coagulative necrosis of some renal tubules and other structures showed tubular dilation. Some renal glomeruli showed shrinkage of their glomerular tuft or an absence with widening of the capsular space. Vacuolation in the epithelial lining of some renal tubules was observed. In the SS+CUR group, the changes were decreased compared with the sodium salicylate-treated rats. The most apparent lesions were vascular congestion and mild epithelial vacuolation, and some renal tubules showed regenerative attempt (Figure 3).

### 3.8. Immunohistochemical Findings

**Figure 4:**
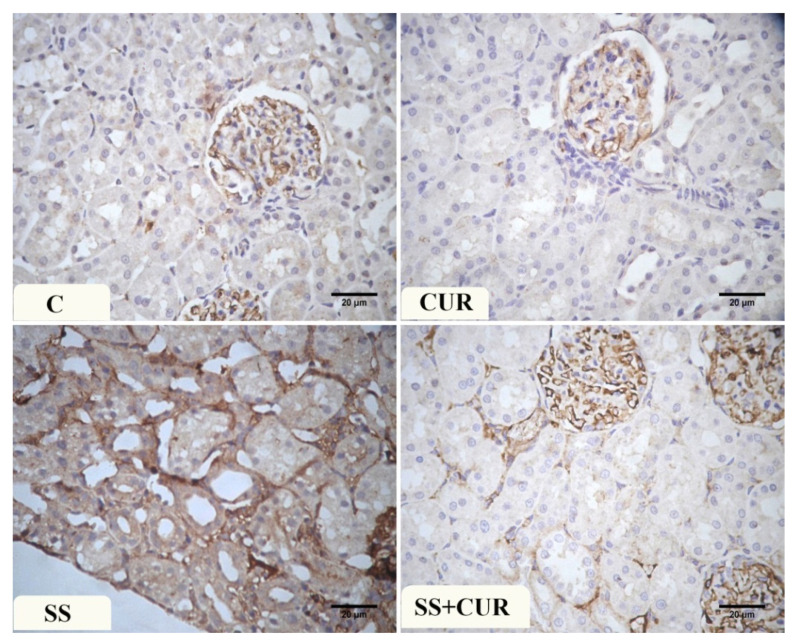
Photomicrograph of the renal tissue sections showing the Caspase -3 immuno-expression in different studied groups: (C) control, (CUR) curcumin, (SS) sodium salicylate, and SS+CUR-treated groups. Magnification ×400.

Immunohistochemical examination of Caspase-3 immuno-expression in the control and CUR group renal tissue revealed a negative to weak reaction in the renal tubules. At the same time, a dark brown color in the glomeruli was detected (Figure 4). In SS-treated rats, a strong positive reaction was mainly present in the cytoplasm of renal tubules. In the SS+CUR-treated group, decreased Caspase-3 immuno-expression was observed and a dark brown color in the glomeruli was noticed.

**Figure 5:**
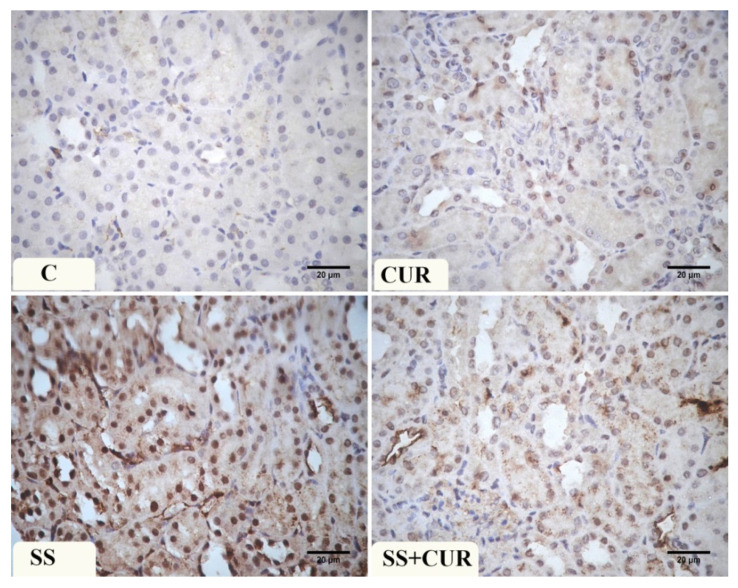
Photomicrograph of the renal tissue sections showing the NF-κB immuno-expression in different studied groups: (C) control, (CUR) curcumin, (SS) sodium salicylate, and SS+CUR-treated groups. Magnification ×400.

The expression of NF-κB of the control and CUR group renal tissue revealed a weak reaction in the control group and a moderate reaction in the CUR group. In SS-treated rats, a strong positive reaction was detected in the nuclei of renal tubules. In the SS+CUR group, the immuno-expression of NF-κB decreased compared to the SS-exposed group (Figure 5).

### 3.9. Correlation Analysis by Principal Components Analysis

**Figure 6:**
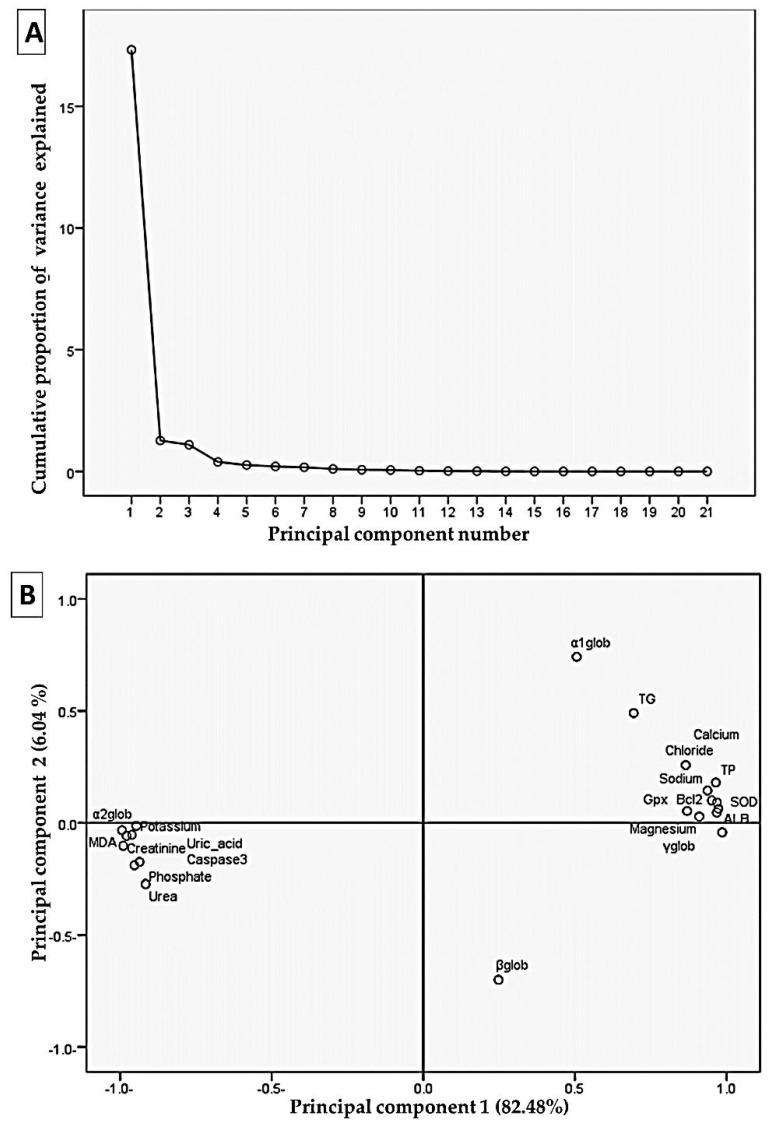
Principal component analysis plot showing the relationships among the estimated variables. (**A**) Cumulative proportion of variance as a function of the number of principal components (PC). (**B**) All of the biochemical indicators plotted as a function of PC1 and PC2, which account for 82.48% and 6.04% of the variance, respectively. TB: total protein; TG: total globulin; ALB: albumin; Gpx: glutathione peroxidase; SOD: superoxide dismutase; and MDA: malondialdehyde.

In this case, the main component analysis tested the relationships between current variables. The first two loading components were outlined as displayed in Figure 6, with nearly 88.52% of the total variation in the experimental data being recorded. The variables grouped narrowly (<90˚) have prominent correlations, and in the loading plot, they have a positive correlation. Subsequently, total protein, magnesium, calcium, albumin, SOD, Bcl2, sodium, γ globulin, chloride, Gpx, and globulin were grouped together and strongly correlated with the first component. These collections were greatly negatively associated with uric acid, MDA, creatinine, phosphate, potassium, caspase3, urea, and α2 globulin.

## 4. Discussion

Salicylate is the main biotransformation product of aspirin [55], one of the most widely used drugs worldwide [56]. At the same time, CUR is widely consumed worldwide as its global market size is expected to reach USD 151.9 million by 2027 [57]. Thus, the outcomes of the simultaneous application of CUR and aspirin or SS is highly warranted.

Initially, the current study showed that neither the individual SS injection nor the oral CUR dose alters the erythrogram elements compared to the control groups. However, the combined SS+CUR administration induced an apparent anemic condition. Similarly, CUR has been reported to worsen anemic condition caused by ecstasy in the male rat, while the CUR alone has not elicit any significant changes in the erythrogram elements [29]. This is the first report that shows the synergistic exacerbating effect of CUR with SS on RBCs. CUR has also been shown to inhibit drug-metabolizing cytochrome P450, UDP-glucuronosyltransferase, and glutathione-S-transferase [58,59,60,61]. The inhibition of such enzymes may result in unwanted increases in the plasma concentrations of certain drugs and may lead to toxicity [61]. Of note, the liver cytochrome’s ability to metabolize xenobiotics has been reported to decline with aging [62,63]. Additionally, most hepatic cytochrome P450 mRNA expressions are reduced with aging [64,65]. Simultaneously, elderly persons extensively use analgesics such as non-steroidal anti-inflammatory drugs and herbal supplements such as CUR [66,67]. Thus, more caution should be taken with the co-use of SS with CUR in elderly persons, and further studies on this point are highly warranted. CUR is an active iron Chelator in vivo and induced anemia in mice fed iron-deficient diets [68]. CUR suppresses the synthesis of hepcidin, one of the iron balance peptides, and has the potential to cause iron deficiency in preclinical iron deficiency [68]. CUR binds ferric iron (Fe^3+^) to form dose-dependent CUR and Fe^3+^ specific composites [69]. This suggests that, particularly in people with iron under optimal conditions, CUR can affect systemic iron metabolism [70].

An obvious thrombocytopenic, leukocytosis, and neutrophilia condition was recorded in SS-injected rats. Similarly, leukocytosis and thrombocytopenia have been previously reported with SS overdose [71]. SS-induced thrombocytopenia could be a consequence of the impaired renal function detected in the SS-injected group [72]. Additionally, neutrophil accumulation may lead to the release of reactive oxygen species (ROS) and may elicit inflammatory responses, which may, in turn, recruit neutrophils [73]. Oppositely, CUR concurrent treatment in SS-injected rats considerably restored the depleted thrombocytes but suppressed the increased leukocytes and neutrophils counts. The CUR antioxidant activity could correct the disturbance of leukocytes and platelet count [74].

Herein, SS substantially increased kidney damage products, including urea, creatinine, and uric acid. Moreover, numerous pathological changes were detected in kidney tissues of SS-injected rats, including vascular congestion, hyalinization of the blood vessel, absence of capsular space, glomerular congestion, coagulative necrosis, congestion of peritubular capillaries, absence of glomerular tuft, tubular dilation, shrinkage of the glomerulus, and vacuolation of the lining epithelium of tubules. Covalent binding of SS or its metabolites with proximal tubular cell mitochondria has been proposed to change these organelles’ function, thereby interfering with the energy supply [5,75,76]. A diminished energy supply in proximal tubular cells can cause active transporter dysfunction or cellular death in more serious conditions [7]. However, a CUR oral dose in SS-injected rats considerably depressed the increases in urea, creatinine, and uric acid and restored normal kidney structure. The protective effect of CUR on kidneys has been evaluated in previous studies with drugs such as gentamicin [77] and cisplatin [78]. This effect can be linked to the antioxidant properties of CUR, since the impairment of the glomerular filter rate may be implied by ROS [79].

In this research, hypoproteinemia, hypoalbuminemia, hypoglobulinemia, and hypo γ globulinemia alongside a decreased albumin/globulin ratio were apparent in SS-injected rats. This might be the outcome of a combination of reduced synthesis and increased degradation of albumin [80]. Additionally, earlier reports confirmed the link between increased α2 globulin and nephrotic syndrome [81]. In contrast, CUR mutual co-treatment in SS-injected rats significantly corrected the altered protein profile reflecting enhanced renal function. Additionally, CUR-associated restoration of total protein, albumin, and globulin could be a consequence of liver protection [82].

The kidneys are the main organ for maintaining the equilibrium and acid balance of different electrolytes in the body [83]. A gradual loss of renal function leads to many adaptable and compensatory renal and extrarenal modifications that allow for homeostasis with moderate glomerular filtration rates to be maintained [84]. With the considerable reduction in glomerular filtration rates, there are usually irregularities in the body’s environment with clinical consequences [85]. In the current study, SS injection for 15 days induced a clear disturbance in the electrolyte balance represented by hyponatremia, hypochloremia, hypocalcemia, and hypomagnesemia but not hyperkalemia and hypophosphatemia. The overall Na body content is the key determinant of extracellular volume, and thus, the sodium balance disturbances could lead to clinical conditions of volume depletion or overload [85]. The kidney’s ability to excrete K is reduced proportionally to glomerular filter loss. The use of drugs that alter the kidneys’ ability to excrete potassium is the leading cause of hyperkalemia [85]. Despite the increase in K level in the SS-treated group, no mortalities were recorded throughout the experiment. In this regard, the effects of hyperkalemia on ECG in the rat may differ from other species. For instance, an earlier report confirmed that, in rats, K concentration could reach 10.8 mmol/L in the blood plasma and returns to normal values within 2 h. [86]. The authors suggested that the redistribution of K between extra- and intracellular fluid plays a major role in the restoration of K homeostasis in rats. Additionally, de Araujo, et al. [87] reported that aging rats adapt to extreme conditions of K deprivation and K loading. Hypokalemia occurs in most patients with moderate to severe salicylate toxicity until serious dehydration, malfunction of the kidney, or rhabdomyolysis result in hyperkalemia [88]. On the other hand, the CUR+SS-treated group showed a significant correction of the electrolytes levels.

Oxidative stress has been implicated in the pathogenesis of various nephrotoxic drugs [89,90]. Moreover, in numerous renal illnesses, apoptosis is frequently involved [91,92]. Herein, SS substantially increased MDA and Caspase-3 amounts in the kidney tissue but depleted SOD, GPx, and BCL-2. Although SS has well-known antioxidant effects at small doses [93], the SS high doses act as a pro-oxidant that promotes cell death. Earlier in vivo and in vitro reports established the oxidative stress implication as a mechanism of salicylates toxicity [94,95]. Moreover, Deng, et al. [96] suggested that high SS concentrations in vitro have resulted in paradoxical superoxide radical upregulation that produced apoptotic consequences. SS-induced apoptosis has been mediated by the activation of p38 mitogen-activated protein kinases, leading to Caspase-3 activation [97]. The oxidative, lipid peroxidative, and apoptotic damage induced by SS can elucidate the pathological disturbances detected during the histopathology of the kidney.

Here, the CUR nephroprotective effect could be greatly related to its strong antioxidant and antiapoptotic activity. Similarly, the nephroprotective effect of CUR via restoration of the antioxidant system and lowered lipid peroxidation in the kidneys of rats has been reported [98]. Additionally, CUR treatment inhibited renal tubular cell apoptosis [30,99]. The antioxidant mechanism of CUR proposed can comprise one or more interactions such as free radical scavenging; neutralization of the oxidative cascade interaction; and the prevention and reduction in the amount of scavenging oxidizing enzymes, such as the cytochrome P450 inhibiting [100], oxygen quenching, and disarming properties of toxicants [101].

Furthermore, the antioxidant mechanism of CUR is due to its unique conjugation structure, comprising two methoxylated phenols and an enol diketone that has a typical radical capability as an antioxidant to break down the chains [102]. CUR therapy induces detoxification enzymes ascribed to free radical scavenging and lysosome enzyme liberating inhibition [103]. CUR helps preserve the cell membrane integrity by preventing peroxidation in the presence of toxicants [104].

The ROS ability to signal cell survival or death appears to be related to gene expression regulation via inhibition or activation of redox responsive transcription factors [105,106]. NF-κB encompasses an inducible transcription factor family that chiefly regulates host inflammatory and immune responses [107]. Several NF-κB activation reports have been recorded in the kidney after exposure to many drugs [108,109]. Herein, an apparent increase in NF-κB immuno-expression in the kidney tissue following SS administration was detected but suppressed following CUR treatment. In this regard, Hoppstädter, et al. [110] suggested that the CUR anti-inflammatory properties are based on the regulation of transcribed factors, growth factors, signal transduction pathways, and inflammatory cytokines by eliminating the signaling of NF-κB.

One of the most frequently used multivariate statistical tools is principal component analysis because it distinguishes samples from two-dimensional projection [111]. In this context, the loading results show a significant correlation between estimated biomarker responses. The amount of total protein and the indicators of oxidative stress (i.e., SOD and GPx) and apoptosis (i.e., Bcl-2) were strongly correlated with various electrolyte disturbances in sodium, chloride, and calcium. In this line, several studies confirm the interplay of oxidative stress and apoptosis in sequencing events leading to kidney dysfunction [112,113].

## 5. Conclusions

Taken together, the collective findings from the current study propose that the SS use at high doses could be nephrotoxic, as manifested by impaired renal function, impaired electrolyte balance, and histopathological perturbations in kidney tissue. This might be mediated mainly through oxidative damage, apoptotic changes, and inflammatory consequences in the kidney. Despite the evident protective role of CUR against SS-induced renal damage, the combined CUR and SS treatment resulted in a significant anemic condition. Further studies on the combined use of CUR and other analgesic drugs, particularly at large doses, are highly recommended.

## Figures and Tables

**Table 1:** Effect of curcumin (CUR) treatment on hematological indices of sodium salicylate (SS)-administered rats for 15 days.

Parameters	Control	OO	CUR	SS	CUR+SS
Relative right kidney weight	0.40 ± 0.04	0.51 ± 0.04	0.46 ± 0.05	0.41 ± 0.10	0.42 ± 0.10
Left relative kidney weight	0.44 ± 0.01	0.48 ± 0.03	0.48 ± 0.08	0.44 ± 0.09	0.44 ± 0.01
Erythrogram					
RBCs (10^6^/mm^3^)	4.35 ± 0.23	4.41 ± 0.65	4.53 ± 0.30	4.40 ± 0.47	2.67 *^#^ ± 0.04
Hb (g/dL)	10.87 ± 0.12	11.47 ± 1.10	11.73 ± 1.47	10.23 ± 1.41	7.20 ^*#^ ± 0.14
PCV (%)	23.07 ± 3.36	22.73 ± 2.82	24.87 ± 2.98	24.47 ± 3.31	14.23 *^#^ ± 0.34
MCV(fl)	52.93 ± 1.89	52.97 ± 1.54	55.40 ± 0.51	55.47 ± 3.14	54.07 ± 0.95
MCH (%)	26.63 ± 0.58	26.70 ± 1.42	25.90 ± 2.01	23.23 * ± 1.00	27.37 ^#^ ± 0.37
MCHC (%)	45.63 ± 2.95	47.57 ± 2.40	43.23 ± 1.84	45.23 ± 3.87	47.93 ± 3.69
Platelets (10^3^/mm^3^)	314.33 ± 23.33	315.33 ± 53.94	565.00 *^#^ ± 134.35	376.67 *± 8.81	534.67 *^#^ ± 66.78
Leukogram					
WBCs (10^3^/mm^3^)	5.10 ± 0.22	5.33 ± 0.21	5.37 ± 0.56	9.40 * ± 0.22	5.23 ^#^ ± 0.53
Neutrophils (10^3^/mm^3^)	19.00 ± 2.45	19.00 ± 2.16	18.67 ± 2.05	24.67 * ± 3.30	19.00 ^#^ ± 4.55
Lymphocytes (10^3^/mm^3^)	69.33 ± 3.86	67.00 ± 2.94	69.67 ± 5.73	69.33 ± 4.03	68.67 ± 6.34
Eosinophils (10^3^/mm^3^)	1.00 ± 0.00	1.33 ± 0.47	1.33 ± 0.47	1.33 ± 0.47	1.00 ± 0.00
Monocytes (10^3^/mm^3^)	11.67 ± 1.25	13.33 ± 2.05	12.67 ± 2.49	11.33 ± 1.25	13.00 ± 0.82
Basophils (10^3^/mm^3^)	0.00 ± 0.00	0.00 ± 0.00	0.00 ± 0.00	0.00 ± 0.00	0.00 ± 0.00

OO: olive oil. Values are represented as the mean ± SD. *n* = 10 replicates/treatment. * Significantly different compared to the control groups at *p* < 0.05. ^#^ Significantly different from the SS-treated group at *p* < 0.05.

**Table 2:** Effect of curcumin (CUR) treatment on biochemical indicators of sodium salicylate (SS) administered rats for 15 days.

Parameters	Control	OO	CUR	SS	CUR+SS
Total protein (g/L)	74.70 ± 3.18	75.23 ± 2.54	74.85 ± 2.96	48.63 * ± 4.86	59.25 *^#^ ± 0.99
Albumin (g/L)	40.80 ± 1.06	41.05 ± 1.51	41.28 ± 1.15	23.08* ± 2.88	30.70 *^#^ ± 3.10
Total globulin (g/L)	33.90 ± 3.64	34.18 ± 3.12	33.58 ± 3.61	25.55 * ± 5.03	28.55 ± 3.00
α1 globulin (g/L)	18.98 ± 3.68	19.30 ± 3.45	18.40 ± 3.81	13.03 ± 6.30	13.45 ± 3.65
α2 globulin (g/L)	6.78 ± 0.21	6.73 ± 0.45	6.90 ± 0.29	9.13 * ± 0.57	8.20 *^#^ ± 0.34
β globulin (g/L)	4.97 ± 0.34	5.06 ± 0.33	5.04 ± 0.12	4.82 ± 0.57	4.99 ± 0.67
γ globulin (g/L)	3.20 ± 0.54	3.08 ± 0.33	3.26 ± 0.46	1.42 * ± 0.38	2.05 *^#^ ± 0.17
Urea (mmol/L)	9.17 ± 1.61	9.39 ± 0.89	9.40 ± 1.38	16.77 * ± 1.77	13.57 *^#^ ± 0.77
Creatinine (umol/L)	72.33 ± 2.17	72.69 ± 3.43	72.26 ± 1.88	107.83 * ± 7.94	90.85 *^#^ ± 3.42
Uric acid (umol/L)	354.70 ± 9.12	355.63 ±9.56	355.49 ± 13.00	727.24 * ± 59.80	544.90 *^#^ ± 14.89
Sodium (mmol/L)	144.33 ± 5.56	140.58 ± 7.15	144.15 ± 11.12	93.66 * ± 8.03	120.54 *^#^ ± 7.39
Potassium (mmol/L)	3.93 ± 0.57	4.53 ± 0.55	4.28 ± 0.70	9.80 * ± 1.14	6.89 *^#^ ± 0.85
Chloride (mmol/L)	91.67 ± 7.04	93.23 ± 4.93	94.87 ± 6.54	65.91 * ± 5.02	84.16 ^#^ ± 6.69
Phosphate (mmol/L)	0.87 ± 0.02	0.85 ± 0.03	0.86 ± 0.05	1.71 * ± 0.15	1.15 *^#^ ± 0.11
Calcium (mmol/L)	2.46 ± 0.20	2.40 ± 0.20	2.46 ± 0.11	1.14 * ± 0.19	1.68 *^#^ ± 0.07
Magnesium (mmol/L)	1.03 ± 0.07	1.03 ± 0.04	1.03 ± 0.07	0.56 * ± 0.07	0.81 *^#^ ± 0.04

OO: olive oil. The values are represented as the mean ± SD. *n* = 10 replicates/treatment. * Significantly different compared to the control groups at *p* < 0.05. ^#^ Significantly different from the SS-treated group at *p* < 0.05.

## Data Availability

All datasets generated for this study are included in the article.
